# Editorial: Factors Influencing Biomarker Range Intervals in Farm Animals

**DOI:** 10.3389/fvets.2020.587741

**Published:** 2020-10-26

**Authors:** Ana M. Gutiérrez, Anna Bassols, Laura Soler, Matilde Piñeiro

**Affiliations:** ^1^Department of Animal Medicine and Surgery, BioVetMed Research Group, University of Murcia, Murcia, Spain; ^2^Department of Biochemistry and Molecular Biology, Faculty of Veterinary Medicine, Autonomous University of Barcelona, Barcelona, Spain; ^3^Institut National de la Recherche Agronomique, Toulouse, France; ^4^Acuvet Biotech, Zaragoza, Spain

**Keywords:** biomarkers, reference values, biological fluids, farm animals, values variation

A wide variety of biomarkers are used in farm animals for different purposes such as diagnostic testing, animal health monitoring, and serological surveillance and management of a farm. However, the presence of non-pathologic factors represents a challenge for valid and reliable biomarker use. These factors will influence reference interval (RI) and interpretation of biomarker test results. They are defined by intra-species genetic variability, and different physiological and environmental conditions, and their impact might be biological and/or analytical. In this special issue, our contributors have addressed some of the problems related with these factors. Harmonization of veterinary biomarker calibration procedures and reagents is also a must for the rationale use of biomarkers, as pointed out in one of the articles.

Yu et al. studied the effects of several variables on the serum biochemical RIs in young animals: age, season of birth and sex in calves and age and sex in piglets. The study comprised unweaned calves (at 24 h and 2, 5, and 7 weeks of age) and piglets from weaning at 21 days old to 35 days of life. In calves, season of birth did not affect the distribution of values of the studied analytes while age-biased differences were noticed. The authors showed that hepatic enzymes, renal markers, antioxidant enzymes (glutatione peroxidase and superoxide dismutase) and some metabolism analytes [non-esterified fatty acids (NEFAs), triglycerides, glucose and total proteins] are increased at 24 h after birth in comparison with other ages (2, 5, and 7 weeks of age). They also showed that, in pigs, the concentrations of cholesterol, triglycerides, and NEFAs are increased just after weaning compared to 28 and 35 days of life and this may be explained by a dietary change from milk, with high fat content, to a solid feed. An increase in cortisol and pig major acute phase protein (Pig-MAP) concentrations was also observed the day after weaning and could be reflecting a stressful condition. Yu et al. also provided the age at which each analyte reached RIs for adult animals.

Henning-Pauka et al. studied how season influences the level of serum acute phase proteins (APP) in piglets in the nursery period (from 3 to 11 weeks of life) and found higher values in autumn compared to winter. The experiment was performed in two indoor-outer nursery compartments of a farrow-to-finishing farm with space for 180 piglets each, in which a new ammonia sensory technology connected to a forced ventilation system was evaluated. No direct influence of ammonia levels on APP concentrations could be confirmed. However, the authors recommend that low ammonium levels and optimal temperatures should be maintained, particularly during periods in which outside temperatures are extreme, to improve production parameters and preserve a good health status of piglets.

Sánchez et al. evaluated how other intrinsic biological factors such as production stage, breed and sex, could influence the RIs of the APP, inflammation biomarker adenosine deaminase (ADA), and total antioxidant capacity (TAC), measured in saliva. The level of these biomarkers in saliva samples from clinically healthy conventional and Iberian pigs were studied in male and female animals at different production stages (post-weaning, nursery, fattening, and finishing). In conventional pigs, sex and production stage influenced the concentration of all the studied biomarkers with higher values in females compared to males at finishing, while the opposite was observed in younger animals, at post-weaning. On the contrary, no sex and production stage differences on the aforementioned biomarkers were observed in Iberian pigs that could be associated with the routine castration of males in this breed. However, ADA levels appeared higher in Iberian males in comparison to females at finishing. Additional studies should be performed to improve our knowledge on saliva biomarkers.

When establishing RIs for biomarkers, it is important to use well-established calibrators to obtain equivalent results between laboratories. Several analytes such as enzymes (enzymatic activity), total protein, ions or metabolites (such as urea or glucose) are measured by chemical reactions which are common for all species and do not present a problem with calibrators. However, protein biomarkers, usually measured by immunoassays, need special attention due to changes in amino acid sequence between the different animal species, or other protein modifications, that result in differences in epitopes recognized by antibodies in the assay. These differences in protein structure or content usually require the use of species-specific calibrators in such biomarker assays. Thus, an international harmonization of veterinary biomarker calibration has been proposed (Eckersall) consisting of two important stages. First, an international consortium would be established to define how to approach the harmonization process and establish a plan of action that should include the following activities: identify biomarkers where standardized calibration is needed, define the protocols for preparation and distribution of reference materials, identify the participating laboratories and find funding opportunities from industry and academia. The working group should include members from the main international/regional societies covering veterinary pathology, and members from industry and experts in the field of study. Second, reference preparations, consisting of a pool of species-specific serum containing the analyte being measured would be prepared and a consensus value assigned for the selected biomarker (Eckersall). The preparations will be available to laboratories and diagnostic kits manufacturers for use as a primary standard to calibrate their working standards.

Serological and molecular tests are the best choice for the detection of a wide variety of infectious diseases using different biological specimens such as serum, saliva, urine, or feces. Some chronic infections result in multisystemic disease that may affect animal production. Therefore, detection of specific chronic infections has been postulated to be a predictor of production losses. Echeverría et al. analyzed the association between seroprevalence of Small Ruminal Lentivirus infection (SRLV) and production losses in two different sheep breeds (Navarra and Latxa Navarra) and production systems (dairy or meat). The authors present antigenic heterogeneity as the main factor influencing the seroprevalence analysis excluding breed, age, production system or animal management effects. However, the possible influence of lentivirus infection on the production status of the studied animals was not evaluated. The results did highlight the importance of combining different detection methods (ELISAs against different antigen preparations and genetic-based test) for determining the SRLV infection status at the herd level in SRLV.

In conclusion, more research must be carried out on the effects of interfering factors before establishing reliable RIs for diagnostic/prognostic analytes in farm animals, especially in alternative body fluids such as saliva. Saliva, as biological fluid, can allow for routine monitoring of animal health status due to its minimally invasive and non-stressful collection method that could be performed by personnel with minimal training. However, the use of saliva biomarkers in the clinical assessment of farm animals is still far from being routinely applied and requires further validation and research, including RI establishment. Continued effort should be invested in the implementation of novel biomarkers in clinical veterinary practice, including assay standardization, publications of RIs with various methods and reagents used globally, and establishment of defined guidelines for result interpretation.

## Author Contributions

AG, AB, LS, and MP contributed to writing this editorial of the Research Topic Factors Influencing Biomarker Range Intervals in Farm Animals.

## Conflict of Interest

The authors declare that the research was conducted in the absence of any commercial or financial relationships that could be construed as a potential conflict of interest.

